# Occupational Exposure to Industrial Dust and Rates of Multiple Sclerosis

**DOI:** 10.1212/WNL.0000000000214762

**Published:** 2026-03-13

**Authors:** Lars Alfredsson, Eva Johansson, Tomas Olsson, Pernilla Stridh, Anna Karin Hedström

**Affiliations:** 1 Institute of Environmental Medicine, Karolinska Institutet, Stockholm, Sweden;; 2 Center for Occupational and Environmental Medicine, Region Stockholm, Sweden; and; 3 Department of Clinical Neuroscience, Karolinska Institutet, Stockholm, Sweden.

## Abstract

**Background and Objectives:**

Exposure to lung irritants such as smoking and organic solvents has been associated with increased risk of multiple sclerosis (MS), particularly among genetically susceptible individuals. The aim of this study was to investigate the association between occupational exposure to industrial dust and MS and to assess potential interactions with smoking and HLA-DRB1*15:01.

**Methods:**

We conducted a Swedish population-based case-control study. Patients with incident MS age 16–70 years were consecutively identified by neurologists at 40 clinics (2005–2015). Eligibility criteria for participants were age 16–70 years, residence in Sweden, and a neurologist-confirmed diagnosis of MS according to the McDonald criteria. Controls without MS were randomly sampled from the national population register using density sampling and frequency-matched to cases on age, sex, and residential area. Occupational dust exposure was assessed using questionnaire data. Logistic regression was used to estimate odds ratios (ORs) and 95% CIs. Additive interactions between dust exposure and smoking and between dust exposure and HLA-DRB1*15:01 were assessed by calculating the attributable proportion (AP) due to interaction. An AP >0 is considered evidence of interaction.

**Results:**

The analytic sample included 2,070 participants and 2,899 controls. The mean age at index was 34.4 years for participants and 35.4 years for controls. Women comprised 72.5% of participants and 75.1% of controls. Industrial dust exposure was associated with increased rate of MS (OR 1.30, 95% CI 1.05–1.63), with a dose-response relationship with duration (OR per 1-year exposure 1.03, 95% CI 1.00–1.06). Evidence of additive interactions was observed between dust exposure and smoking (AP 0.32, 95% CI 0.03–0.62) and between dust exposure and HLA-DRB1*15:01 (AP 0.25, 95% CI 0.002–0.52). Participants who smoked, were exposed to dust, and carried the HLA-DRB1*15:01 allele had an 11-fold increased rate of MS (OR 11.1, 95% CI 5.7–21.9), compared with those without any of these risk factors.

**Discussion:**

Occupational dust exposure was associated with increased rate of MS, particularly in combination with smoking and HLA-DRB1*15:01, suggesting joint effects of occupational, environmental, and genetic risk factors. The reliance on self-reported occupational histories and potential residual confounding are important limitations. Further studies are warranted to clarify underlying mechanisms and to inform preventive strategies.

## Introduction

Multiple sclerosis (MS) is a chronic, immune-mediated disease of the CNS, driven by a complex interplay between genetic predisposition and environmental factors.^1^ The strongest genetic associations with MS are found within the HLA complex, with carriage of the HLA-DRB1*15 allele increasing the risk of MS approximately 3-fold.^2,3^ Environmental risk factors for MS include Epstein-Barr virus infection,^4,5^ low vitamin D levels/low sun exposure,^5,6^ adolescent obesity,^5,7^ and exposure to lung irritants such as smoking,^8^ organic solvents,^9^ and air pollution.^10^ The risk associated with these lung irritants is significantly amplified in individuals with a genetic susceptibility to MS, as evidenced by synergistic effects between each irritant and the major genetic risk factors.^11-13^ No previous study has specifically evaluated exposure to occupational dust in relation to MS. We hypothesized that dust exposure, as another inhaled irritant, may similarly influence MS susceptibility.

In a Swedish population-based case-control study, we sought to investigate the influence of exposure to occupational dust on MS rate and to explore potential interactions with smoking and the HLA-DRB1*15 allele.

## Methods

### Study Design and Study Participants

This study was a part of the Epidemiological Investigation of Multiple Sclerosis (EIMS),^14^ a population-based case-control study involving the general Swedish population aged 16–70 years. Incident cases of MS were consecutively identified by treating neurologists at 40 neurology units across Sweden, including all university clinics, during the recruitment period April 2005 to April 2015. Participants were invited to participate in EIMS at the time of diagnosis. Eligibility criteria for participants were age 16–70 years, residence in Sweden, and a neurologist-confirmed diagnosis of MS according to the McDonald criteria.^15,16^ For each participant, 2 control participants without MS were randomly selected from the national population register using density sampling, frequency-matched to the participant by age in 5-year strata, sex, and residential area. All invitees received a standardized questionnaire and an invitation to provide a blood sample. Participants who returned questionnaires with missing or ambiguous responses were contacted by study staff to clarify and complete items. During the study period, 2,807 participants and 5,950 control participants completed a standardized questionnaire that collected detailed information on environmental exposures and lifestyle-related factors. The response rate was 93% among participants and 73% among control participants. Blood samples were made available from 2,070 participants and 2,899 control participants, all of whom were included in this study (eFigure 1).

### Standard Protocol Approvals, Registrations, and Patient Consents

The study was approved by the Regional Ethical Review Board at Karolinska Institute (reference number 2004-252/1-4 and 2013/1691-32) and was conducted in accordance with the principles of the Declaration of Helsinki. All participants provided informed consent.

### Data Sources and Variable Ascertainment

Age, sex, and residential area were obtained from the national population register and used for density sampling and frequency matching of control participants to participants. Age was grouped in 5-year strata, and residential area at the county level in Sweden. All other variables were ascertained from the standardized questionnaire, and classical 4-digit HLA alleles were imputed from MS replication chip genotypes using HLA*IMP:02 software.^17,18^

For each participant, the time of initial MS symptom onset was used to estimate the disease onset, with the year of symptom onset designated as the index year. The corresponding control participants were assigned the same index year as their matched participant. Participants provided information on occupational exposures by indicating whether they had been exposed to any of the following types of industrial dust: wood dust, asbestos dust, textile dust, and stone dust (eMethods). Stone dust exposure included responses to separate questions on stone dust, stone crushing, and rock drilling, which were combined into a single exposure category. Those who reported exposure were asked to specify the start and end year for each exposure period. Total duration (years) was calculated as the sum of all reported periods occurring before the index year. Overlapping periods were merged to avoid double counting. Participants who reported occupational exposure to any of the specified types of dust before the index year were classified as exposed.

Smoking status was determined by asking participants about their current and past smoking habits. Participants who smoked at the index year were defined as current smokers, whereas those who did not smoke were categorized as nonsmokers. The rationale for this dichotomizing is based on previous findings that the effect of smoking on MS risk, and its interaction with genetics, slowly abates after smoking cessation.^14^

Ancestry was categorized as Nordic vs non-Nordic. Past infectious mononucleosis was self-reported as yes, no, or unsure, and modeled as a 3-level category. Adolescent body mass index was categorized as underweight, normal weight, overweight, or obesity, as defined by the World Health Organization. Alcohol consumption at diagnosis was categorized into nondrinkers, low alcohol consumption (<50 g per week for female participants and <100 g per week for male participants), moderate alcohol consumption (50–108 g per week for female participants and 100–168 g per week for male participants), and high alcohol consumption (>108 g per week for female participants and >168 g per week for male participants). Physical activity at diagnosis was categorized into low (physical activity without sweating for less than 2 hours per week), moderate (physical activity without sweating for at least 2 hours per week), moderate/high (regularly exercising for at least 30 minutes 1–2 times per week), or high (regularly exercising for at least 30 minutes at least 3 times per week). Based on 3 questions regarding sun exposure at diagnosis (sunbathing in Sweden, traveling to sunnier countries, and use of sunbeds), where each answer alternative was given a number ranging from 1 (the lowest exposure) to 4 (the highest exposure), we constructed an index by summing the scores (range 3–12) and dichotomized at the median into high vs low exposure.

Regarding genetic risk factors, participants were categorized based on the presence or absence of HLA-DRB1*15:01, DRB1*03:01, DRB1*08:01, DRB1*13:03, HLA-A*02:01, B*44:02, B*38:01, B*55:01, DQA1*01:01, DQB1*03:02, and DQB1*03:01, respectively. Gene-environment interaction analyses were prespecified for DRB1*15:01. Remaining alleles were included as adjustment covariates.

### Statistical Analysis

The occurrence of MS in participants who reported occupational exposure to industrial dust was compared with that of participants who had never been exposed, by calculating odds ratios (ORs) with 95% CIs using unconditional logistic regression models.^19^ Across tables we report 2 adjustment sets. The first model adjusted for the matching factors and ancestry. In a second model, we additionally included past infectious mononucleosis, adolescent body mass index, smoking, alcohol consumption, physical activity, and sun exposure. The interaction analyses further included the following HLA alleles which have been independently associated with risk of MS^2^: DRB1*03:01, DRB1*13:03, DRB1*08:01, A*02:01, B*44:02, B*38:01, B*55:01, DQA1*01:01, DQB1*03:02, and DQBI*03:01. When a variable was used for stratification, it was not included as an adjustment covariate in the corresponding stratified model.

A trend analysis for dose-response with years of dust exposure was performed by entering duration as a continuous variable in the logistic regression model, with never exposed coded as 0, without a separate binary exposure term. We report the OR per 1-year increase with 95% CI. Linearity on the log-odds scale was evaluated by restricted spline terms and a likelihood-ratio test.

Potential interactions between dust exposure and both current smoking and the HLA-DRB1*15:01 allele were assessed by calculating the attributable proportion (AP) because of interaction together with a 95% CIs, derived using the delta method.^20,21^ Let OR_01_, OR_10_, and OR_11_ denote ORs for A-only exposure, B-only exposure, and both exposures (vs OR_00_ = 1). We computed the relative excess risk because of interaction (RERI) as RERI = OR_11_ − OR_10_ − OR_01_ + 1, and AP = RERI/OR_11_. The AP between 2 interacting risk factors reflects the joint effect beyond the sum of the individual effects, where a value greater than zero indicates the presence of interaction, suggesting that they are involved in the same pathway to disease.

Although our main hypothesis was that dust exposure in general, as a lung irritant, may influence the risk of MS, we also studied the specified types of dust separately. For descriptive purposes, we visualized the distribution of exposure duration among exposed participants using a kernel density plot by case-control status. Finally, we used conditional logistic regression for sensitivity analyses, conditioning on the matched case-control sets. Otherwise, the model specification was identical to the primary analyses. All analyses were conducted using SAS version 9.4.

### Data Availability

Anonymized data underlying this article will be shared on reasonable request from any qualified investigator that wants to analyze questions that are related to the published article.

## Results

Our analysis included 2,070 participants and 2,899 control participants matched by age, sex, and residential area. The mean age at onset was 34.4 years, and the median duration from disease onset to diagnosis was 1.0 year. Almost all participants were recruited within 1 year of diagnosis, and the questionnaires were completed after a median of 2.0 years after disease onset. The characteristics of participants and control participants, stratified by exposure to occupational dust, are presented in Table 1.

**Table 1:** Characteristics of Participants and Control Participants

	Total		Dust exposure		No dust exposure	
	Participants	Controls	Participants	Controls	Participants	Controls
N	2,070	2,899	195	210	1,875	2,689
Dust exposure, y, median (IQR)	—	—	5 (1–40)	5 (1–36)	—	—
Age at index, y, mean (SD)	34.4 (10.7)	35.4 (10.8)	35.2 (10.6)	37.8 (11.1)	34.4 (10.7)	35.2 (10.7)
Women, n (%)	1,500 (72.5)	2,177 (75.1)	101 (51.8)	118 (56.2)	1,399 (74.6)	2,059 (76.6)
Nordic, n (%)	1,690 (81.6)	2,301 (79.4)	161 (82.6)	166 (79.1)	1,529 (81.6)	2,135 (79.4)
Past IM, n (%)	355 (17.3)	290 (10.0)	22 (11.5)	24 (11.5)	333 (17.9)	266 (9.9)
No past IM, n (%)	1,510 (73.4)	2,392 (82.8)	151 (78.7)	166 (79.4)	1,359 (72.9)	2,226 (83.1)
Unsure, n (%)	192 (9.3)	206 (7.1)	19 (9.9)	19 (9.1)	173 (9.3)	187 (7.0)
Never smoking	976 (47.1)	1,611 (55.6)	71 (36.4)	104 (49.5)	905 (48.3)	1,507 (56.0)
Current smoking	653 (31.6)	685 (23.6)	74 (38.0)	53 (25.2)	579 (30.9)	632 (23.5)
Past smoking	441 (21.3)	603 (20.8)	50 (25.6)	53 (25.2)	391 (20.9)	550 (20.5)
Alcohol, n (%)	1,432 (69.2)	2,103 (72.5)	134 (68.7)	158 (75.2)	1,298 (69.2)	1,945 (72.3)
Underweight	268 (13.0)	392 (13.5)	15 (7.7)	18 (8.6)	253 (13.5)	374 (13.9)
Normal weight	1,447 (69.9)	2,200 (75.9)	137 (70.3)	156 (74.3)	1,310 (69.9)	2,044 (76.0)
Overweight	259 (12.5)	250 (8.6)	29 (14.9)	30 (14.3)	230 (12.3)	220 (8.2)
Obesity	96 (4.6)	57 (2.0)	14 (7.2)	6 (2.9)	82 (4.4)	51 (1.9)
Low sun exposure, n (%)	767 (37.1)	893 (30.8)	82 (42.1)	82 (39.1)	685 (36.5)	811 (30.2)

Abbreviations: IM = infectious mononucleosis; IQR = interquartile range.

Counts/percentages are based on variable-specific nonmissing denominators. The smoking variable captures cigarette smoking; participants reporting exclusive past use of cigarillos, cigars, or pipe (3 participants, 1 control participant) were coded as nonsmokers.

Exposure to any occupational dust was reported by 195 (9.4%) participants and 210 (7.2%) control participants. Subtype distributions were generally comparable between participants and control participants (eTable 1). We observed a significant association between dust exposure and increased rate of MS (OR 1.30, 95% CI 1.05–1.63) (Table 2). A significant trend with duration of exposure was also noted (OR per year 1.03, 95% CI 1.00–1.06), indicating a 3% increase in rate for each additional year of exposure. Among exposed participants, the distribution of duration was right-skewed and broadly similar between groups (eFigure 2). No deviation from linearity was detected.

**Table 2:** OR With 95% CI of Developing MS for Individuals Occupationally Exposed to Industrial Dust, Compared With Non-Exposed Individuals

Exposure	Paticipants/controls	OR (95% CI)^a^	OR (95% CI)^b^
Any industrial dust			
No	1,875/2,689	1.0 (reference)	1.0 (reference)
Yes	195/210	1.33 (1.08–1.63)	1.30 (1.05–1.63)
Specific types of industrial dusts			
Wood			
No	1,989/2,809	1.0 (reference)	1.0 (reference)
Yes	81/90	1.22 (0.89–1.67)	1.22 (0.88–1.70)
Asbestos			
No	2,024/2,856	1.0 (reference)	1.0 (reference)
Yes	46/43	1.47 (0.96–2.24)	1.39 (0.89–2.17)
Stone			
No	2,049/2,889	1.0 (reference)	1.0 (reference)
Yes	21/10	2.75 (1.87–5.87)	2.31 (1.03–5.18)
Textile			
No	1,991/2,810	1.0 (reference)	1.0 (reference)
Yes	79/89	1.31 (0.96–1.78)	1.38 (0.99–1.91)

Abbreviations: MS = multiple sclerosis; OR = odds ratio.

a Adjusted for age, sex, residential area (according to study design), and ancestry.

b Adjusted for age, sex, residential area (according to study design), ancestry, past IM, smoking, alcohol consumption, body mass index, and sun exposure habits.

The combination of smoking and dust exposure interacted to further elevate the rate of MS, with an AP of 0.32 (95% CI 0.03–0.62), indicating that about 32% of the excess rate among participants exposed to both factors is attributable to their interaction (Table 3). The association with dust exposure was more pronounced among participants with a genetic susceptibility to MS, as evidenced by a significant interaction between dust exposure and the DRB1*15:01 allele (AP 0.25, 95% CI 0.002–0.52) (Table 4).

**Table 3:** OR With 95% CI of Developing MS for Individuals With Different Combinations of Smoking Status at Disease Onset and Occupational Exposure to Industrial Dust

Smoking	Dust exposure	Cases/controls	OR (95% CI)^a^	OR (95% CI)^b^	AP
Nonsmoker	No	1,296/2,057	1.0 (reference)	1.0 (reference)	
Nonsmoker	Yes	121/157	1.22 (0.95–1.56)	1.19 (0.91–1.54)	
Current smoking	No	579/632	1.45 (1.27–1.65)	1.43 (1.24–1.64)	
Current smoking	Yes	74/53	2.17 (1.51–3.12)	2.38 (1.62–3.49)	0.32 (0.03–0.62)

Abbreviations: AP = attributable proportion; MS = multiple sclerosis; OR = odds ratio.

a Adjusted for age, sex, residential area (according to study design), and ancestry.

b Adjusted for age, sex, residential area (according to study design), ancestry, past IM, alcohol consumption, body mass index, sun exposure habits, HLA-DRB1*15:01, DRB1*03:01, DRB1*13:03, DRB1*08:01, A*02:01, B*44:02, B*38:01, B*55:01, DQA1*01:01, DQB1*03:02, and DQB1*03:01.

**Table 4:** OR With 95% CI of Developing MS for Individuals With Different Combinations of HLA-DRB1*15:01 Status and Occupational Exposure to Industrial Dust

HLA-DRB1*15:01	Dust exposure	Participants/controls	OR (95% CI)^a^	OR (95% CI)^b^	AP
Noncarrier	No	851/1,938	1.0 (reference)	1.0 (reference)	
Noncarrier	Yes	92/153	1.31 (0.99–1.73)	1.28 (1.00–1.74)	
Carrier	No	1,024/751	4.00 (3.42–4.68)	4.03 (3.44–4.73)	
Carrier	Yes	103/57	5.59 (3.91–7.99)	5.76 (4.02–8.27)	0.25 (0.002–0.52)

Abbreviations: AP = attributable proportion; MS = multiple sclerosis; OR = odds ratio.

a Adjusted for age, sex, residential area (according to study design), and ancestry.

b Adjusted for age, sex residential area (according to study design), ancestry, past IM, smoking, alcohol consumption, body mass index, sun exposure habits, HLA-DRB1*03:01, DRB1*13:03, DRB1*08:01, A*02:01, B*44:02, B*38:01, B*55:01, DQA1*01:01, DQB1*03:02, and DQB1*03:01.

When we categorized the participants based on DRB1*15:01 status, current smoking status, and dust exposure, the triple-exposed had an OR of 11.1 (95% CI 5.7–21.9) compared with those with none of the exposures (Table 5). The results were consistent when using conditional logistic regression (eTables 2 and 3).

**Table 5:** OR With 95% CI of Developing MS for Individuals With Different Combinations of HLA-DRB1*15:01 Status, Smoking Status, and Occupational Exposure to Industrial Dust

DRB1*15:01	Smoking	Dust	Participants/controls	OR (95% CI)^a^	OR (95% CI)^b^
−	−	−	594/1,474	1.0 (reference)	1.0 (reference)
−	−	+	57/112	1.23 (0.88–1.73)	1.16 (0.82–1.63)
−	+	−	257/464	1.37 (1.14–1.64)	1.34 (1.11–1.61)
−	+	+	35/41	2.11 (1.32–3.36)	2.13 (1.32–3.43)
+	−	−	702/583	3.07 (2.65–3.56)	3.81 (3.20–4.54)
+	−	+	64/45	3.54 (2.38–5.28)	4.66 (3.08–7.06)
+	+	−	322/168	4.85 (3.93–6.00)	5.97 (4.73–7.53)
+	+	+	39/12	8.22 (4.24–15.9)	11.1 (5.65–21.9)

Abbreviations: MS = multiple sclerosis; OR = odds ratio.

a Adjusted for age, sex, residential area (according to study design), and ancestry.

b Adjusted for age, sex, residential area (according to study design), ancestry, past IM, alcohol consumption, body mass index, sun exposure habits, HLA-DRB1*03:01, DRB1*13:03, DRB1*08:01, A*02:01, B*44:02, B*38:01, B*55:01, DQA1*01:01, DQB1*03:02, and DQB1*03:01.

## Discussion

In this study, we observed a significant association between occupational exposure to dust and an increased rate of developing MS. Synergistic effects between dust exposure and both smoking and the HLA-DRB1*15:01 allele were observed. Specifically, the combination of dust exposure, smoking, and genetic susceptibility conferred a markedly elevated rate of MS, underscoring the importance of considering environmental and genetic factors together when assessing MS risk.

The observed association between dust exposure and MS rate aligns with previous research linking other lung irritants, such as smoking, air pollution, and organic solvents, to an increased risk of MS.^8-14^ These exposures have shown comparable effects sizes, with smoking and organic solvents associated with approximately 50% increased risk,^8-11^ and more modest associations observed for air pollution.^12^ Inhaled occupational exposures have also been associated with an increased risk of other autoimmune and inflammatory diseases, such as systemic lupus erythematosus, systemic sclerosis, and rheumatoid arthritis (RA).^22-25^ Many inhalable occupational exposures have been found to interact with both smoking and RA-associated genetic risk factors, substantially increasing RA risk in genetically susceptible individuals.^25^

Our findings support the hypothesis that exposure to lung irritants may contribute to the development of MS, potentially through mechanisms involving inflammation and immune activation in the respiratory tract.^11^ Lung irritants might trigger autoaggressive T cells in the lungs or induce modifications in self-proteins, making them cross-reactive with CNS antigens.^26-28^ These activated T cells or modified proteins may subsequently migrate to the CNS, leading to inflammation and the onset of MS.^29^ The significant interaction between dust exposure and smoking further suggests that these environmental factors may exacerbate each other's effects, resulting in a synergistic increase in MS rate.

The observed interaction between dust exposure and the HLA-DRB1*15:01 allele in relation to MS rate may be attributable to the classical function of class II molecules, which have distinct preferences for peptide binding. In individuals with the *HLA-DRB115:01* allele, chronic lung inflammation triggered by lung irritants may facilitate the activation of autoimmune CD4^+^ T cells. This activation could then contribute to the development of MS.

Beyond susceptibility, the observed associations with inhaled irritants have potential implications for prognosis. Postdiagnosis smoking has been consistently associated with a worse course.^30,31^ By the same rationale, continued occupational exposure to occupational dust after onset could plausibly exacerbate disease activity and accelerate disability accumulation through persistent pulmonary inflammation and immune activation. Testing this hypothesis will ideally require longitudinal studies relating postdiagnosis dust exposure to disability progression. If confirmed, exposure reduction could be incorporated as a secondary-prevention target in MS care.

The strengths of this study include its large sample size, the population-based design, and the ability to examine gene-environment interactions in a well-characterized cohort. The availability of both genetic and detailed environmental data allowed for a robust analysis of interactions between multiple risk factors. However, several limitations should be considered. The number of participants exposed to specific types of dust was relatively small, limiting the statistical power to detect significant associations for individual dust types. In addition, the self-reported nature of the exposure data may introduce recall bias, although the use of detailed questionnaires and the inclusion of newly diagnosed cases likely mitigated this risk to some extent. There were no differences in professions or lines of work between participants and control participants among those who reported dust exposure, indicating a similar reporting pattern. Furthermore, participants who reported dust exposure provided occupational titles consistent with jobs where dust exposure is highly probable, such as construction workers, carpenters, textile factory workers, and insulation installers. The comparison group was defined as individuals who reported no exposure to any of the specified dust exposures. Exposure misclassification may have occurred because of recall error and unmeasured dust types. Given the incident participant recruitment, identical questionnaires for participants and control participants, and no reason to expect case-status–dependent reporting, any misclassification is most plausibly nondifferential and would, on average, attenuate associations. Nonetheless, departures from nondifferential assumptions cannot be totally excluded.

A potential selection bias may arise when recruiting participants and control participants, particularly if the likelihood of participation is related to exposure status. However, given that the Swedish public health care system provides equal, free access to medical services for all citizens, nearly all participants of MS are referred to neurologic units. The high response rates (93% for participants and 73% for control participants) suggest that selection bias is likely modest. The prevalence of smoking among the control participants, a useful indicator of lifestyle factors, was consistent with that of the general population in similar age groups.^32^ In addition, there were no significant differences with respect to age, sex, smoking habits, or dust exposure between those who provided blood and those who did not. Despite adjusting for key confounders, the possibility of residual confounding cannot be entirely ruled out. Finally, we did not have sufficient statistical power to analyze current vs past dust exposure at index, although this distinction may be relevant.

In conclusion, our study indicates that occupational exposure to industrial dust is associated with an increased rate of MS, particularly when combined with smoking and genetic susceptibility. These findings contribute to the growing body of evidence linking lung irritants to MS and underscore the importance of considering gene-environment interactions in MS etiology.

## Glossary

AP: attributable proportion
EIMS: Epidemiological Investigation of Multiple Sclerosis
MS: multiple sclerosis
OR: odds ratio
RA: rheumatoid arthritis
RERI: relative excess risk due to interaction
